# Electroacupuncture stimulation modulates functional brain connectivity in the treatment of pediatric cerebral palsy: a case report

**DOI:** 10.3389/fpsyt.2024.1392958

**Published:** 2024-04-22

**Authors:** Zongbo Sun, Chenglin Li, Laixin Sun, Wenwen Yang, Xueli Qu, Yuanyuan Li, Xiao Duan, Fengyu Guo, Xuejing Sun, Mingzhu Yang, Tong Qi, Longyun Zhu, Shuai Wang, Yu Xia, Yanan Du, Shuhui Luo, Lingling Li, Yu Gu, Yaya Wang, Li Yang

**Affiliations:** ^1^ School of Medicine, Liao Cheng University, Liaocheng, Shandong, China; ^2^ Department of Rehabilitation, Maternal and Child Health Hospital, Dongchangfu, Liaocheng, China; ^3^ Department of Radiology, Maternal and Child Health Hospital, Dongchangfu, Liaocheng, China; ^4^ Medical Genetics Laboratory, Maternal and Child Health Hospital, Dongchangfu, Liaocheng, China

**Keywords:** electroacupuncture, meta-analysis, language, brain functional connectivity, pediatric cerebral palsy

## Abstract

**Background:**

Pediatric cerebral palsy (CP) is a non-progressive brain injury syndrome characterized by central motor dysfunction and insufficient brain coordination ability. The etiology of CP is complex and often accompanied by diverse complications such as intellectual disability and language disorders, making clinical treatment difficult. Despite the availability of pharmacological interventions, rehabilitation programs, and spasticity relief surgery as treatment options for CP, their effectiveness is still constrained. Electroacupuncture (EA) stimulation has demonstrated great improvements in motor function, but its comprehensive, objective therapeutic effects on pediatric CP remain to be clarified.

**Methods:**

We present a case of a 5-year-old Chinese female child who was diagnosed with CP at the age of 4. The patient exhibited severe impairments in motor, language, social, and cognitive functions. We performed a 3-month period of EA rehabilitation, obtaining resting state functional magnetic resonance imaging (rs-fMRI) of the patient at 0 month, 3 months and 5 months since treatment started, then characterized brain functional connectivity patterns in each phase for comparison.

**Results:**

After a 12-month follow-up, notable advancements were observed in the patient’s language and social symptoms. Changes of functional connectivity patterns confirmed this therapeutic effect and showed specific benefits for different recovery phase: starting from language functions then modulating social participation and other developmental behaviors.

**Conclusion:**

This is a pioneering report demonstrating the longitudinal effect of EA stimulation on functional brain connectivity in CP patients, suggesting EA an effective intervention for developmental disabilities (especially language and social dysfunctions) associated with pediatric CP.

## Introduction

Pediatric cerebral palsy (CP) is a central nervous system disorder syndrome characterized by postural and motor dysfunction, mainly caused by non-progressive brain injury during the immature stage of brain development within one month after birth (1, 2). It has complex pathogenic factors and is often accompanied by symptoms such as intellectual disability, behavioral abnormalities, and visual, auditory, language disorders, posing a serious threat to the physical and mental health of the affected child. Currently, rehabilitation therapy remains the primary treatment option for pediatric CP (3). Traditional rehabilitation training aimed at improving movement disorders by reducing spasticity and deformities, but the therapeutic effect on the central nervous system functions is not satisfactory. Novel interventions are needed to further enhance the quality of life in these children.

Studies have shown that acupuncture combined with standardized rehabilitation can greatly improve the motor functions of children with CP (4–6). By stimulating specific parts of the hairline area of the head with millineedles, acupuncture exerts a beneficial influence in activating blood, removing stasis, filling the medullary sea, nourishing the brain orifices, and enhancing brain intelligence, which holds positive significance in enhancing brain neural function. Electroacupuncture (EA) stimulation involves the application of electrical currents to specific acupoints on the body following acupuncture, and has been widely used in the treatment of various neurological disorders. It is convenient to use and can effectively compensate for the disadvantages of long needle retention time for children and inconvenient needle operation for doctors. However, the comprehensive therapeutic effects of EA on the brain functions (in addition to dyskinesia) of pediatric CP remain to be clarified.

Currently, the rehabilitation assessment methods for CP mainly include gross motor function assessment (GMFM) (7), Gesell Developmental Scales (GDS) (8), and fine motor function assessment for infants and young children (FMFM) (9). However, considering the complex clinical symptoms of pediatric CP, and the difficulty for infants and young children to cooperate with the examination work, using only clinical scales to evaluate the efficacy is not reliable enough. Resting state functional magnetic resonance imaging (rs-fMRI) is an important imaging method for studying brain functions and central nervous system pathological mechanisms (10–12), which can provide more objective evaluation of the therapeutic effect of EA.

The effective implementation of EA stimulation in this case presents a novel viewpoint and promising advancement in the management of pediatric CP, as supported by fMRI analysis.

## Methods and results

**Figure 1:**
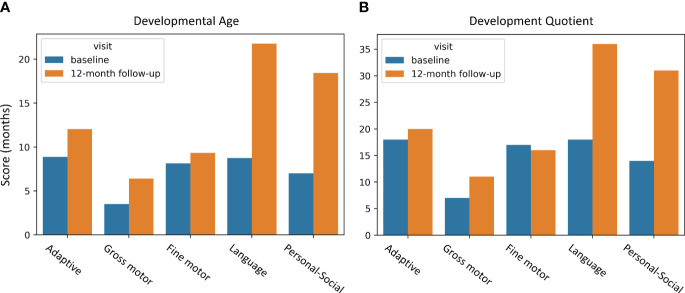
GDS behavior improvements due to EA intervention. **(A)** DA improvements; **(B)** DQ improvements. The horizontal axis represents behaviors, and the vertical axis indicates assessment scores. The blue bar displays the baseline assessment before EA, and the yellow bar is the 12-month follow-up assessment after EA. GDS, Gesell Developmental Scales; DA, developmental age; DQ, developmental quotient.

We present a case of a 5-year-old Chinese female child who was born with neonatal asphyxia and intracranial hemorrhage due to abnormal fetal position, umbilical cord around neck, and insufficient amniotic fluid during pregnancy. The patient was diagnosed as CP at the age of 4, and denied family history. This patient experienced developmental delay since birth, with symptoms of difficulty in centering the head, difficulty in symmetrical limbs, inability to roll over, tendency to lean back in a sitting position, and high muscle tone in the limbs. Gesell Development Scales assessment indicated extremely severe developmental delay in all of the 5 zones: adaptive behavior (Developmental Age (DA): 8.88 months, Developmental Quotient (DQ): 18 months), gross bodily control (DA: 3.51 months, DQ: 7 months), fine motor coordination (DA: 8.13 months, DQ: 17 months), language (DA: 8.75 months, DQ: 18 months), and personal-social behavior (DA: 7 months, DQ: 14 months) at baseline (**Figure 1**). The patient had no history of organic brain disease, head injury, or head surgery, and had not received any rehabilitation or medication treatment.

Considering the patient’s young age, we performed relatively safe, non-invasive electroacupuncture intervention to improve her neural development as well as minimize trauma and medication side effects to the greatest extent possible. Acupuncture acupoints of the patient’s functional impairment including Si Shen Cong plus Bai Hui, Three Wisdom Needles (Shen Ting, Ben Shen), 1/5 above the anterior oblique line of the parietal and temporal regions were selected. During the operation, family members fixed the child’s head and limbs. 75% alcohol cotton balls were used to sterilize the skin of the acupuncture points, and the disposable acupuncture needles (0.30 mm × 25 mm) were used for acupuncture. The needle was inserted under the skin at an angle of 30° to the scalp until the tip of the needle reached the lower layer of the cap like aponeurosis. Then, continued to insert the needle parallel to the scalp until the depth of the needle was 3/4 of the needle body. The acupuncture was performed using a rapid twisting technique (200 revolutions per minute), and after obtaining qi (i.e. achieving a sensation or response, such as numbness and swelling at the acupuncture site from the patient, indicating the proper placement and stimulation of the acupuncture needle), intermittent waves (2-15 Hz) were transmitted on the needle, adjusting the intensity to a level that the child could tolerate, lasting for 30 minutes. The patient was treated once a day, with continuous treatment for 6 days and 1 day of rest. The total treatment consisted of three courses, with each course lasting for 20 days. The specific acupoints stimulated in each course are illustrated in **Table 1**.

**Table 1:** Specific acupoints stimulated in each course.

	Course 1	Course 2	Course 3
Bai Hui	Y	–	–
Shen Ting	Y	–	–
Si Shen Cong	–	Y	Y
Ben Shen	–	Y	Y
1/5 above the anterior oblique line of the parietal and temporal regions	Y	Y	Y

Y is short for yes, meaning that the acupoint was stimulated during the course of treatment.

These modifications resulted in a general amelioration of the patient’s condition (**Figure 1**). Notably, the language and personal-social behavior were improved significantly with the improvement ratio of 148.8%, 163.29% for developmental age (DA), and 100%, 121.43% for development quotient (DQ), respectively, according to the Gesell Development Scales (GDS). Other assessment scores were also improved to different extents, including adaptive behavior (DA: 35.47%, DQ: 11.11%), gross bodily control (DA: 82.34%, DQ: 57.14%), fine motor coordination (DA: 14.76%, DQ: -5.88%).

To investigate the underlying alterations in brain functions associated with these improvements induced by EA, we obtained resting-state functional magnetic resonance imaging (rs-fMRI) of the patient 0 month, 3 months and 5 months since the EA treatment started. The data were acquired on a 1.5T MRI system (Philips) with head coil. BOLD sequence was performed with the imaging protocol: matrix = 256 × 256, slice thickness = 4mm, slice spacing = 1mm, field of view (FOV) = 180-230 mm, repetition time (TR) = 2000ms, echo time (TE) = 30ms, isotropic voxel size = 3.5mm. The rs-fMRI images were preprocessed by fMRIPrep20.1.3 (13). In brief, functional data were processed by slice timing correction using 3dTshift from AFNI (14), and motion correction using FSL-mcflirt (15). After the preliminary preprocessing, regional time series were obtained by extracting the Schaefer atlas (16) with 100 cortical parcels matched to seven functional brain networks defined by Yeo (17), and Pearson’s Correlation Coefficient was used to measure functional connectivity. The leading eigenvector of the functional connectivity was extracted to characterize the main functional connectivity pattern (18–21).

**Figure 2:**
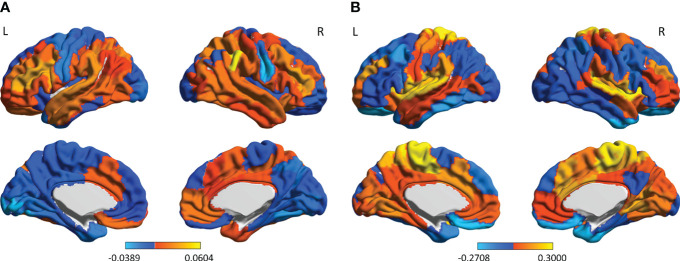
The FC-changes patterns during the two recovery phases of EA treatment: **(A)** the 0-3-month phase; and **(B)** the 3-5-month phase. The color bar indicates the magnitude of FC changes in each brain area. L, left hemisphere; R, right hemisphere.

We computed the main functional connectivity patterns for different recovery phase (0-month, 3-month and 5-month), respectively, and measured the difference between phases (**Figure 2**). The maximum FC changes occurred in the parietotemporal and inferior frontal areas including the opercular inferior frontal, the triangular inferior frontal, the postcentral fissure, the SupraMarginal, the heschl, the superior temporal and the temporal pole, exhibiting remarkable left hemisphere lateralization in the 0-3-month phase; while the 3-5-month alterations of FC showed symmetry with a greater changing amplitude and wider spatial distribution, mainly involved in areas of the sensorimotor network, the salience/ventral attention network, the limbic system, and the Dorsal Attention Network, including the superior orbitofrontal, the rolandic operculum, the rectus gyrus, the insula, the paracentral lobule, the heschl gyrus and the superior temporal.

**Figure 3:**
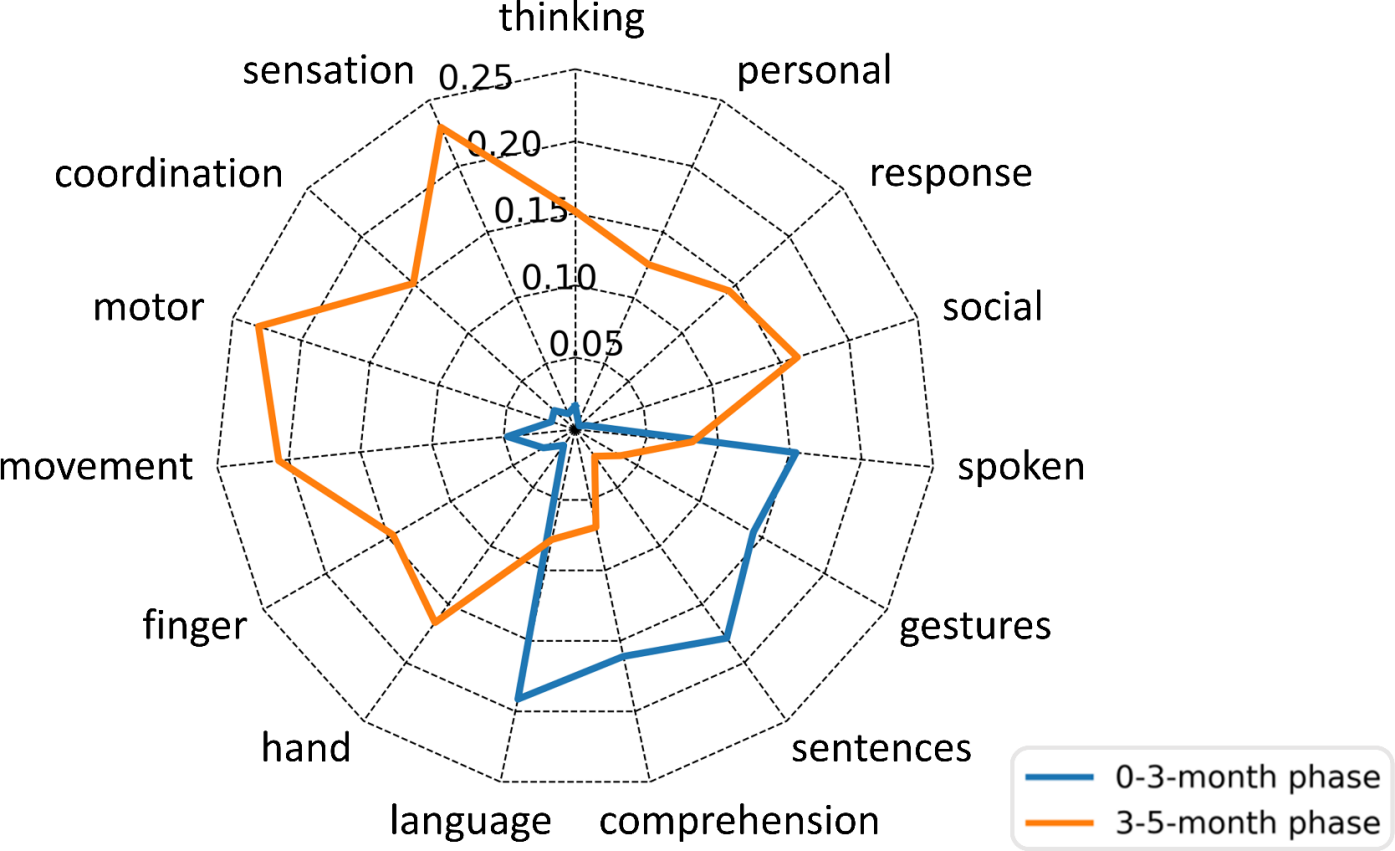
Probabilistic functional profile of the two phases’ FC-changes patterns based on the Neurosynth meta-analysis. Scores indicate the Pearson’s correlations between each FC-changes pattern and Neurosynth topics. The blue line represents the 0-3-month phase; the yellow line represents the 3-5-month phase.

In order to assess the functional significance of the FC changes caused by EA, we mapped the difference patterns to distinct behavioral profiles using Neurosynth (22, 23), which enables functions to be decoded from spatial expression of brain activity based on a meta-analysis of 507891 brain activations reported in 14371 studies (www.neurosynth.org). This yielded a functional profile of the two phases’ FC-changes patterns (0-3-month and 3-5-month) across 15 selected topics related to the Gesell Developmental Scales (8): thinking, sensation, coordination, motor, movement, finger, hand, language, comprehension, sentences, gestures, spoken, social, response and personal. Phases’ FC changes showed distinct functional signatures (**Figure 3**). The 0-3 month phase has a strong association with language (Pearson correlation coefficient r = 0.1913), comprehension (r = 0.161), sentences (r = 0.179), gestures (r = 0.1428) and spoken (r = 0.1542); while the 3-5 month phase has pattern that map onto social (r = 0.1622), response (r = 0.1438), personal (r = 0.1252), thinking (r = 0.1513), sensation (r = 0.2295), coordination (r = 0.1513), motor (r = 0.2312), movement (r = 0.207), finger (r = 0.1462), and hand (r = 0.1656) functions. It indicated that EA enhanced language functions of the pediatric CP patient first, then acting on motor, personal-social and adaptive behavior, which explained the developmental improvements according to GDS.

## Discussion

CP affects approximately 1.5 to 3 out of every 1000 live births, making it one of the leading causes of childhood disabilities. The fundamental pathophysiology involves damage to the developing brain during the prenatal and neonatal stages (1, 3, 24, 25). While the initial brain damage in CP is non-progressive, affected children may experience various secondary conditions that can impact their functional abilities to varying degrees (3, 26). Rehabilitation therapy, such as physiotherapy (27, 28), occupational therapy (27) and use of adaptive equipment and orthoses (29, 30), is the main clinical intervention to impact on the neuroplasticity of the developing brain thus improving patients’ symptoms (3). However, traditional rehabilitation training often requires a significant amount of time to achieve therapeutic effects. Other interventions, such as pharmacologic botulinum toxin injections, intrathecal baclofen, and selective dorsal rhizotomy, carry risks and may have detrimental effects on physical functions (2, 3).

In this report, we introduced a relatively conservative, safe and effective treatment, electroacupuncture, for pediatric CP, and described its efficiency on a case with severe developmental delay. After EA intervention, the patient’s symptoms were effectively improved, especially the language and personal-social behaviors, which was associated with remodeling of functional brain connectivity. We found that FC of the left inferior frontal and parietotemporal areas related to language functions changed most in the initial phase of recovery. It is in line with other brain function studies suggesting language centers to be left hemisphere lateralization advantage (for right-handed) (31–33). In addition, these areas (the opercular inferior frontal, the triangular inferior frontal, the postcentral fissure, the SupraMarginal, the heschl and the superior temporal) overlapped with Broca’s area (34, 35) and Wernicke’s area (36, 37), which are recognized as important language areas (38, 39). During the later phase of recovery, more extensive FC changes involved with motor, personal-social and adaptive functions emerged, among which the most modified areas: the superior orbitofrontal, the gyrus rectus, the insula, and the superior temporal are considered to be associated with self-awareness and social cognition (40–43). These results demonstrate that EA stimulation is beneficial for functional reconstruction of brain network and promotes normal neural development in pediatric CP.

The research on the application of EA in the treatment of pediatric CP is still in its preliminary stages. Available research has shown that EA is more effective than traditional body acupuncture in relieving the gastrocnemius muscle tone in spastic CP of children (44), and the curative effect of scalp acupuncture is better than that of conventional rehabilitation in the treatment of pediatric CP (6). Many studies have suggested acupuncture to be a safe intervention for pediatric CP with no serious adverse event reported (6, 45, 46). Although there are no reports on the safety of EA in the treatment of CP yet, studies have confirmed its great safety on other neurological disorders (47–49). There are few studies comparing EA with other pharmacologic or invasive intervention. However, considering their side effects (such as the destructive effect of selective posterior rhizotomy and the risks of botulinum toxin and intrathecal baclofen on respiratory depression and catheter failure (2)) and long-term expenses on medication maintenance, EA is safer and more suitable for children with reasonable cost-effectiveness.

In light of our findings, we recommend EA as a complementary treatment modality incorporated alongside conventional rehabilitation training to enhance the overall effectiveness of the CP rehabilitation program, especially for children with severe language and personal-social symptoms. This requires additional training and expertise in administering electroacupuncture for clinicians. Collaborative efforts between acupuncturists and rehabilitation specialists would also be essential in developing integrated treatment protocols. Besides, some children with CP might have a fear of needles or may experience discomfort during the procedure. It is important for clinicians to address these concerns through patient education, creating a supportive and reassuring environment, and employing strategies to enhance patient comfort. Further research and clinical trials are needed to explore the long-term effects, optimal treatment protocols, and potential benefits of integrating EA into existing rehabilitation programs for pediatric CP.

As CP is a complex condition influenced by various factors, each patient with CP has a unique clinical profile, including differences in the severity and location of brain lesions, comorbidities, and developmental trajectories. These individual heterogeneities can introduce variability in the response to EA. Although EA treatment achieved good efficacy on this patient, the lack of control groups and large-scale samples made it challenging to differentiate the specific effects of EA from other personalized factors. However, successful application of EA in this case provides clues and hypotheses for subsequent research, and suggests it is worthy of further validation through large-scale experiments to obtain conclusions with greater external validity and generalizability.

In conclusion, EA stimulation in this case of severe pediatric CP demonstrated a remarkably positive therapeutic effect on developmental disabilities, especially the language and personal-social behaviors, involving neural remodeling of functional brain connectivity in the corresponding functional areas in sequence. These results highlight the practicality and efficacy of EA in treating pediatric CP, and emphasize the significance of conducting extensive clinical trials on CP-EA intervention.

## Data availability statement

The raw data supporting the conclusions of this article will be made available by the authors, without undue reservation.

## Ethics statement

The studies involving humans were approved by the ethics committee of Liaocheng Dongchangfu district Maternal and Child Health Hospital, China. The studies were conducted in accordance with the local legislation and institutional requirements. Written informed consent for participation in this study was provided by the participants’ legal guardians/next of kin. Written informed consent was obtained from the individual(s), and minor(s)’ legal guardian/next of kin, for the publication of any potentially identifiable images or data included in this article.

## Author contributions

ZS: Writing – original draft. CL: Writing – original draft. LS: Writing – original draft. WY: Writing – review & editing, Data curation. XQ: Writing – review & editing, Data curation. YL: Writing – review & editing, Formal analysis. XD: Writing – review & editing, Methodology. FG: Writing – review & editing, Methodology. XS: Writing – review & editing, Resources. MY: Writing – review & editing, Resources. TQ: Writing – review & editing, Resources. LZ: Writing – review & editing, Resources. SW: Writing – review & editing, Data curation. YX: Writing – review & editing, Validation. YD: Writing – review & editing, Resources. SL: Writing – review & editing, Resources. LL: Writing – review & editing, Software. YG: Writing – review & editing, Resources. YW: Writing – review & editing, Resources. LY: Writing – review & editing, Writing – original draft, Supervision, Funding acquisition.
